# Experimental determination of translational start sites resolves uncertainties in genomic open reading frame predictions – application to *Mycobacterium tuberculosis*

**DOI:** 10.1099/mic.0.022889-0

**Published:** 2009-01

**Authors:** Katherine L. Smollett, Amanda S. Fivian-Hughes, Joanne E. Smith, Anchi Chang, Tara Rao, Elaine O. Davis

**Affiliations:** Division of Mycobacterial Research, MRC National Institute for Medical Research, The Ridgeway, Mill Hill, London NW7 1AA, UK

## Abstract

Correct identification of translational start sites is important for understanding protein function and transcriptional regulation. The annotated translational start sites contained in genome databases are often predicted using bioinformatics and are rarely verified experimentally, and so are not all accurate. Therefore, we devised a simple approach for determining translational start sites using a combination of epitope tagging and frameshift mutagenesis. This assay was used to determine the start sites of three *Mycobacterium tuberculosis* proteins: LexA, SigC and Rv1955. We were able to show that proteins may begin before or after the predicted site. We also found that a small, non-annotated open reading frame upstream of Rv1955 was expressed as a protein, which we have designated Rv1954A. This approach is readily applicable to any bacterial species for which plasmid transformation can be achieved.

## INTRODUCTION

Mapping the protein translational start site for genes is essential to define the protein sequence, intergenic distances and upstream DNA regions which may contain regulatory motifs. Correct identification of intergenic distances is important for predicting operons and small untranslated RNAs (Carter *et al.*, 2001; Moreno-Hagelsieb & Collado-Vides, 2002). Therefore correct identification of translational start sites is necessary for understanding both protein function and transcriptional regulation (Makita *et al.*, 2007; Rison *et al.*, 2007).

There are many programs for determining open reading frames (ORFs) across genomes using mathematical, probabilistic models. One of the most widely used for prokaryotic genomes is glimmer, which tends to use the first possible translation initiation codon (ATG, GTG or TTG) for a particular gene, giving the longest possible ORF (Delcher *et al.*, 1999). Other approaches take into account factors such as the location of ribosome-binding sites and protein sequence comparisons (Besemer *et al.*, 2001; Makita *et al.*, 2007; Nielsen & Krogh, 2005). However, due to the larger amount of validated data, these methods are often optimized for *Escherichia coli* and the accuracy of these predictions, particularly with regard to the translational start site, can vary for different genomes (Makita *et al.*, 2007). The inaccuracy of ORF and translational start site predictions is particularly a problem with GC-rich genomes, such as that of *Mycobacterium tuberculosis*, due to the more frequent occurrence of the GTG start codon, which is often more common than stop codons (TAA, TGA, TAG), resulting in more possible start codons for each stop codon than in AT-rich organisms (Nielsen & Krogh, 2005). Therefore, the annotated start codons for genes are not necessarily correct, and where more than one possible start codon occurs it is important to verify these experimentally.

Experimental methods for determining translational start sites of proteins are cumbersome. Edman degradation is a commonly used approach. In this method the amino acids of a protein or peptide are removed one by one to determine its sequence (Edman, 1950). More recently an experimental approach based on mass spectrometry was developed and applied to *M. tuberculosis* (Rison *et al.*, 2007). In this method cellular proteins are separated by 2D electrophoresis, individual protein spots are trypsin-digested, and the peptide masses are determined by mass spectrometry. The translational start site is then determined by comparing the actual mass of the N-terminal peptide to those generated *in silico* for alternative start sites. This technique can be used as a high-throughput method to assess the translational start sites of many proteins as part of the determination of the bacterial proteome. However, it is likely that many proteins will be missed by this technique as many proteins do not form visible spots on 2D gels and the N-terminal peptides generated may not be detected by mass spectrometry.

Therefore, we devised a simple approach for determining translational start sites of individual proteins of interest using a combination of epitope tagging and frameshift mutagenesis. It has previously been demonstrated that the C-terminal portion of the product of the human proto-oncogene c-*myc* can be used as an epitope tag for the detection of recombinant proteins in mycobacteria (Spratt *et al.*, 2005). In this assay for determining protein translational start sites, the genes of interest along with their promoter regions were cloned into a vector to give an in-frame C-terminal *myc* tag sequence and single-residue deletions were created between potential start codons, avoiding any potential promoter regions or ribosome-binding sites. Therefore, if the deletion occurs downstream of the actual translational start codon then the resulting protein, including the tag, would be out of frame. Deletions upstream of the start codon will not affect the frame of the protein. Whether the protein is in-frame or out of frame can be detected by the presence or absence of the Myc-tagged protein in cell-free extract. This assay was used to determine the start sites of the *M. tuberculosis* proteins LexA, SigC and Rv1955. We demonstrated that proteins may begin before or after the predicted site. We were also able to use this technique to show that a small, non-annotated ORF was expressed as a protein.

## METHODS

### Bacterial strains and media.

*Escherichia coli* strain DH5*α* (Invitrogen) was used for all plasmid construction and strain XL1-Blue (Stratagene) was used for site-directed mutagenesis (Sambrook *et al.*, 1989). The mycobacterial strains used were *Mycobacterium smegmatis* mc^2^155 (Snapper *et al.*, 1990) and *M. tuberculosis* H37Rv. *E. coli* was grown in Luria–Bertani (LB) broth or on LB agar plates, while *M. smegmatis* and *M. tuberculosis* were grown in modified Dubos medium (Difco) supplemented with 4 % albumin and 0.2 % (w/v) glycerol or on Difco Middlebrook 7H11 agar (Beckton Dickinson) plates supplemented with 4 % albumin and 0.5 % (w/v) glycerol. *E. coli* and *M. smegmatis* liquid cultures were grown at 37 °C with shaking at 225 r.p.m. and *M. tuberculosis* was grown at 37 °C in a rolling incubator at 2 r.p.m. All procedures with live *M. tuberculosis* were carried under ACDP containment level 3 conditions. Where appropriate 50 μg kanamycin ml^−1^ (for *E. coli*) or 25 μg kanamycin ml^−1^ (for mycobacteria) was added to the media.

### Plasmid construction.

The plasmids used and their construction are described in Table 1, and the primers used in this study are listed in Table 2. All site-directed mutagenesis was performed using the Quickchange site-directed mutagenesis (SDM) kit (Stratagene). All constructs were verified by DNA sequencing.

### Preparation of cell-free extracts and Western blotting.

Mycobacterial cultures were grown to mid-exponential phase (OD_600_ 0.6) unless otherwise stated; the bacteria were harvested, washed three times in PBS and cell-free extract prepared as described previously (Davis *et al.*, 2002). In the case of *M. tuberculosis* extracts, the supernatant was filtered through a low-binding Durapore 0.22 μm membrane filter (Ultrafree-MC; Millipore) to ensure complete removal of bacteria before removal from containment facilities. Where mitomycin C induction was required, mycobacterial cultures were grown to early exponential phase (OD_600_ 0.3) and were then split into two; one culture was induced with 0.02 μg mitomycin C ml^−1^ and the other was an uninduced control. Both were then incubated at 37 °C for 24 h before harvesting and preparing cell-free extract as above.

Cell-free extracts were separated by SDS-PAGE and the proteins were electroblotted onto a PVDF membrane using a semi-dry blotter (Hybaid) at 1 mA cm^−2^ for 1 h. Equal loading of the cell-free extract was confirmed by Coomassie blue staining of an identical gel. Western blotting was performed using rabbit anti-Myc (A-14, Santa Cruz) as the primary antibody, at a 1 : 2000 dilution, and anti-rabbit conjugated to horseradish peroxidase (Dako) as the secondary antibody, at a 1 : 5000 dilution. The blot was developed using ECL Western blotting detection reagents (GE Healthcare) according to the manufacturer's instructions.

### Preparation of RNA and RT-PCR.

RNA was prepared from mycobacteria using the FastRNA Pro Blue kit (Qbiogene). Contaminating DNA was removed using the TURBO DNA-free kit (Ambion) and first-strand DNA synthesis was performed using Superscript II (Invitrogen) with random primers (Promega). To confirm the presence of the specific transcript, PCR was then performed on the cDNA using the reverse primer MycRTR with forward primers LexARTF, SigCRTF, JORTF or Rv1955RTF (Table 2) as appropriate. To assess whether ORF2 is co-transcribed with Rv1955–Rv1957 within *M. tuberculosis* H37Rv, PCR was performed on cDNA using forward primer ORF2F with reverse primers ORF2-Rv1955R, ORF2-Rv1956R or ORF2-Rv1957R (Table 2) as appropriate. PCR was also performed on RNA without reverse transcriptase to control for DNA contamination.

### 5′ RACE transcriptional start site mapping.

5′ RACE System for Rapid Amplification of cDNA Ends (Version 2.0; Invitrogen) was performed to map the transcriptional start site of Rv1955 according to the manufacturer's guidelines, using primers Rv1955 GSP1, GSP2 and GSP3. cDNA was tailed at the 3′ ends with poly-cytosine and transcriptional start sites were identified at the junction with the poly-cytosine tail.

### Sequence analysis.

DNA analysis and potential ORF identification was performed using SeqBuilder (dnastar) and Translator (http://www.fr33.net/translator). Sequence analysis was performed using clustal w (http://www.ebi.ac.uk/Tools/clustalw2/index.html) and blast (http://www.ncbi.nlm.nih.gov/blast/Blast.cgi).

## RESULTS

### Determination of the translational start site of LexA

**Fig. 1. f1:**
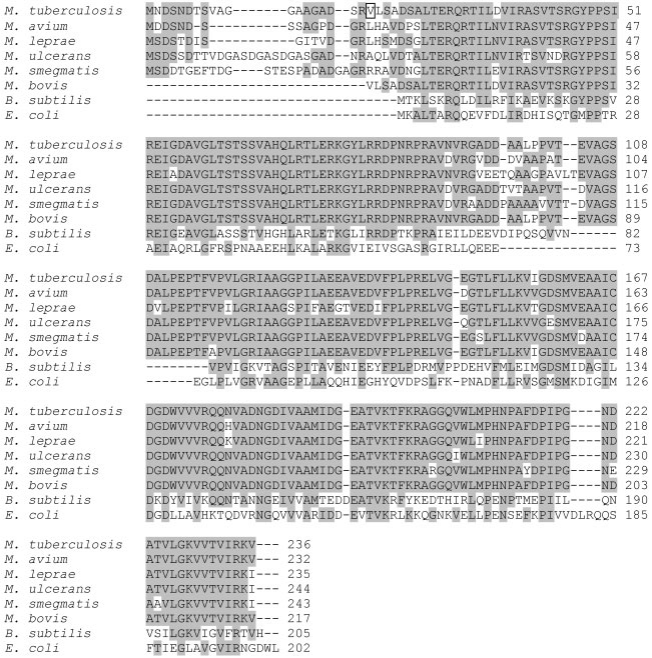
Comparison of *M. tuberculosis* LexA with that of other mycobacterial species. *M. tuberculosis* strain H37Rv LexA (accession no. CAB09461) including 19 aa upstream of the annotated start site was compared to LexA from *M. avium* strain 104 (ABK66179), *M. leprae* strain TN (CAC31384), *M. ulcerans* strain Agy99 (ABL05545), *M smegmatis* strain mc^2^155 (ABK70187) and *M bovis* BCG Pasteur strain 1173P2 (CAL72721) as well as LexA from *B. subtilis* (AAA22573) and *E. coli* (AAA24067) using clustal w. Conserved residues are shaded; the amino acid corresponding to the annotated translational start site of *M. tuberculosis* LexA is boxed.

The translational start site for LexA was initially assigned by aligning the predicted *M. tuberculosis* LexA sequence with other available LexA homologues, primarily those of *E. coli* and *Bacillus subtilis* (Movahedzadeh *et al.*, 1997). However, more recently, the sequences of LexA from other mycobacterial species have become available (Brosch *et al.*, 2007; Cole *et al.*, 2001; Stinear *et al.*, 2007). Alignment of the various predicted mycobacterial LexA protein sequences, including those of *M. avium*, *M. leprae*, *M. ulcerans*, *M. smegmatis* and *M. bovis* (Fig. 1), revealed that all of the mycobacterial LexA sequences examined, with the exception of the *M. bovis* protein, included 15–26 extra amino acids at the N terminus compared to *M. tuberculosis* LexA as predicted from the annotated translational start site. The additional amino acid sequence resulting from translation of *M. tuberculosis* LexA from an upstream alternative start site showed a strong similarity with the amino acid sequences of the N-terminal region of the other mycobacterial LexAs, indicating the possibility that the *M. tuberculosis* LexA is longer than previously thought. Therefore we decided to ascertain the *M. tuberculosis* LexA translational start site experimentally.

**Fig. 2. f2:**
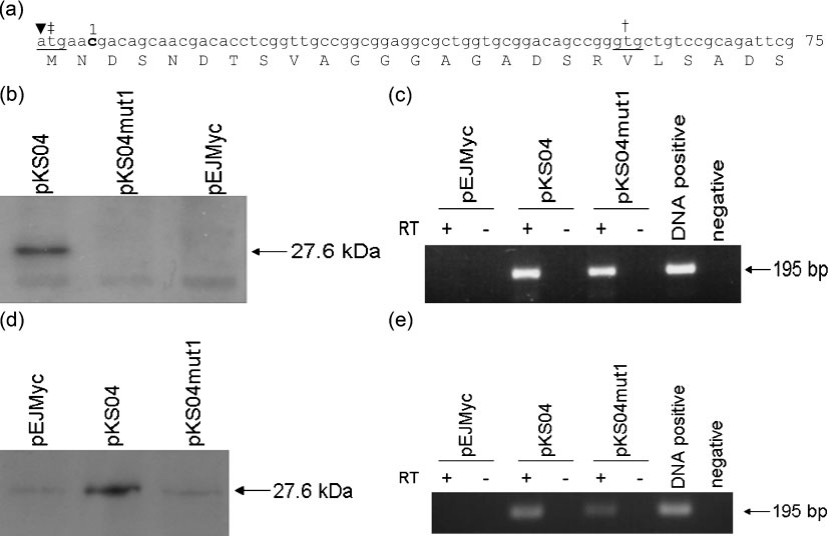
Identification of the LexA translation start site. (a) The nucleotide sequence, and corresponding amino acid translation, of the beginning of the *lexA* gene including 57 bp upstream of the annotated start codon. Two possible start codons are underlined; the annotated start site is indicated by †, the experimentally determined start codon by ‡ and the transcriptional start site by ▾. To determine the translational start codon, a single-residue deletion (bold and numbered) was created between the two possible start codons. (b) Western blot analysis of cell-free extracts of *M. smegmatis* containing pEJMyc, pKS04 or pKS04mut1. A 27.6 kDa band corresponding to LexA-Myc can be detected from strains containing pKS04 but not pEJMyc or pKS04mut1. (c) RT-PCR analysis of RNA extracts of *M. smegmatis* containing pEJMyc, pKS04 or pKS04mut1. A 195 bp band indicating the presence of *lexA-myc* transcript was detected from strains containing pKS04 and pKS04mut1 but not strains containing pEJMyc or the RNA without reverse transcriptase (RT) controls. (d) Western blot analysis of cell-free extracts of *M. tuberculosis* containing pEJMyc, pKS04 or pKS04mut1. (e) RT-PCR analysis of RNA extracts of *M. tuberculosis* containing pEJMyc, pKS04 or pKS04mut1.

The *lexA* gene, including its promoter region, was cloned into pEJMyc to give pKS04, giving LexA with an in-frame C-terminal Myc tag. LexA contains two possible start codons (Fig. 2a[Fig f2]): the annotated start site and a potential alternative start site 57 bases upstream coincident with the transcriptional start site. Therefore, one residue was deleted between these possible start codons to give pKS04mut1 (Fig. 2a[Fig f2]). The plasmids pKS04, pKS04mut1 and the pEJMyc vector were then transformed separately into *M. smegmatis* strain mc^2^155, a commonly used model strain for *M. tuberculosis*. Cell-free extract was prepared from each strain and the presence or absence of Myc-tagged protein was determined by Western blotting (Fig. 2b[Fig f2]). A 27.6 kDa band corresponding to Myc-tagged LexA was detected in cell-free extract from *M. smegmatis* containing pKS04 but not in cell-free extract from *M. smegmatis* containing pKS04mut1 or pEJMyc. We tested for the presence of a specific mRNA transcript of *myc*-tagged *lexA*, to determine that the inability to detect Myc-tagged protein in these strains was due to lack of translation of the tag and not a lack of transcription. RT-PCR was performed using the primers LexARTF and MycRTF, which amplify 195 nucleotides at the 3′ end of the transcript, including the *myc* sequence. The presence of *lexA-myc* transcript was detected in RNA isolated from *M. smegmatis* containing pKS04 and pKS04mut1 but not pEJMyc or the reverse-transcriptase-negative controls (Fig. 2c[Fig f2]). pEJMyc, pKS04 and pKS04mut1 were then transformed into *M. tuberculosis* strain H37Rv to confirm that the translation start site is the same in both mycobacterial species. The presence of LexA-Myc was detected in cell-free extract from *M. tuberculosis* expressing pKS04 but not pKS04mut1 or pEJMyc (Fig. 2d[Fig f2]). The presence of RNA transcript for *lexA-myc* was confirmed by RT-PCR as above (Fig. 2e[Fig f2]). Therefore the translational start site for LexA is upstream of residue 1, indicating that LexA is not translated from the annotated start site and is instead translated from a start codon 57 bp further upstream at the transcriptional start site.

### Determination of the translational start site of SigC

The predicted translational start site for the sigma factor SigC in *M. tuberculosis* differs in the annotation for strain H37Rv compared to that for strain CDC1551 (Camus *et al.*, 2002; Fleischmann *et al.*, 2002). Although the two nucleotide sequences are identical, the annotated translational start of *sigC* for strain CDC1551 is 378 bp upstream of that of strain H37Rv, giving an extra 126 aa at the N terminus. Starting at this start site would also result in a 244 bp overlap with *blaC*, encoded on the opposite strand. Therefore, we decided to determine which of these translational start sites was correct.

**Fig. 3. f3:**
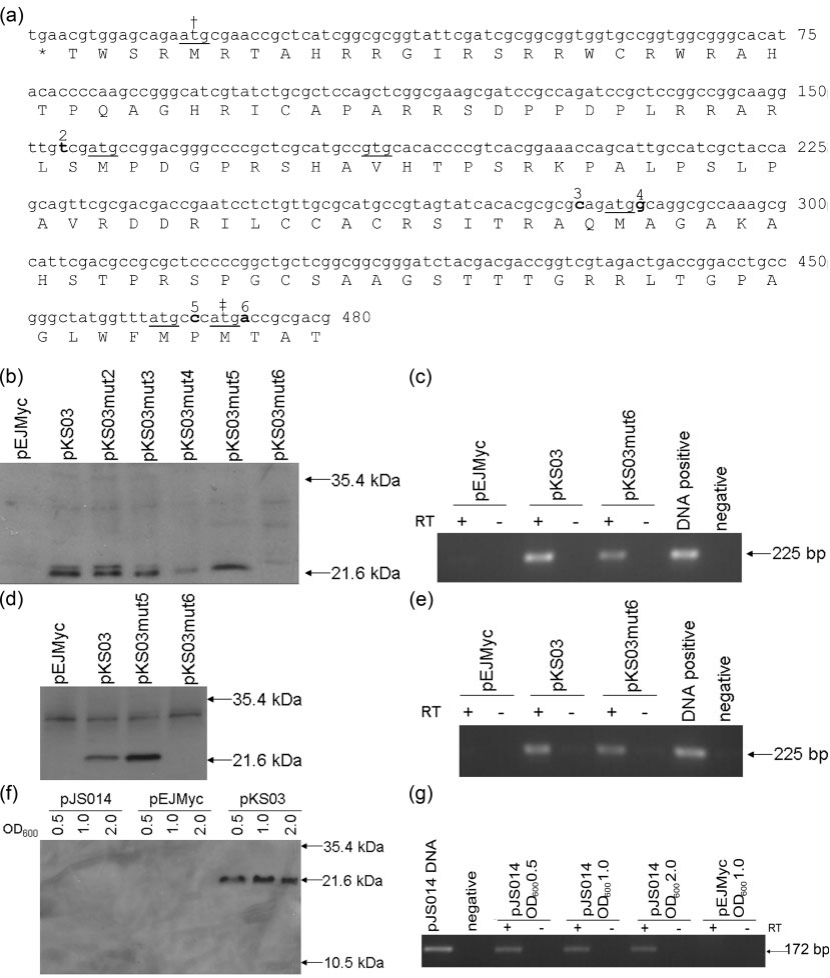
Identification of the SigC translational start site. (a) The nucleotide sequence, and corresponding amino acid translation, of the beginning of the *sigC* gene including 393 bp upstream of the H37Rv annotated start codon. Six possible start codons are underlined; the annotated start site of *M. tuberculosis* strain CDC1551 is indicated by †, that of H37Rv by ‡. To determine the translational start codon, single-residue deletions (bold and numbered) were created between potential start codons. (b) Western blot analysis of cell-free extracts of *M. smegmatis* containing pEJMyc, pKS03, pKS03mut2, pKS03mut3, pKS03mut4, pKS03mut5 or pKS03mut6. A 21.6 kDa band corresponding to the smallest SigC-Myc can be detected from strains containing pKS03, pKS03mut2, pKS03mut3, pKS03mut4 and pKS03mut5 but not pEJMyc or pKS03mut6. No larger SigC-Myc of 35.4 kDa can be detected in any strain. (c) RT-PCR analysis of RNA extracts of *M. smegmatis* containing pEJMyc, pKS03 or pKS03mut6. A 225 bp band indicating the presence of *sigC-myc* transcript was detected from strains containing pKS03 and pKS03mut6 but not strains containing pEJMyc or the RNA without RT controls. (d) Western blot analysis of cell-free extracts of *M. tuberculosis* containing pEJMyc, pKS03, pKS03mut5 or pKS03mut6. (e) RT-PCR analysis of RNA extracts of *M. tuberculosis* containing pEJMyc, pKS03, or pKS03mut6. (f) Western blot analysis of cell-free extracts of *M. tuberculosis* containing pEJMyc, pJS014 or pKS03 at different stages of growth indicated by OD_600_. A 21.6 kDa band corresponding to SigC-Myc can be detected from strains containing pKS03 at all growth phases. No larger SigC-Myc of 35.4 kDa with pKS03 or 10.5 kDa truncated SigC-Myc with pJS014 can be detected at any growth stage examined. (g) RT-PCR analysis of RNA extracts of *M. tuberculosis* containing pEJMyc or pJS014 at different growth stages.

The *sigC* gene and its promoter region was cloned into pEJMyc to give pKS03, containing *sigC* with an in-frame C-terminal Myc tag. There are five alternative start codons upstream of the annotated *sigC* translational start site. Single base pair deletions were created between potential start codons to give plasmids pKS03mut2, pKS03mut3, pKS03mut4, pKS03mut5 and pKS03mut6, where 2–6 refers to the residue deleted (Fig. 3a[Fig f3]). pKS03, and its derivatives, were transformed into *M. smegmatis* mc^2^155. Cell-free extract was prepared from each strain and the presence or absence of Myc-tagged protein was determined by Western blotting (Fig. 3b[Fig f3]). Due to the large number of amino acids between potential start codons, SigC-Myc was expected to be detected between 35.4 kDa and 21.6 kDa, for the largest and smallest potential ORFs respectively. Myc-tagged SigC was detected in extract from *M. smegmatis* containing pKS03, pKS03mut2, pKS03mut3 pKS03mut4 and pKS03mut5 at 21.6 kDa, but was not detected in extract from *M. smegmatis* containing pKS03mut6 or pEJMyc. The presence of mRNA transcript for *sigC-myc* in *M. smegmatis* carrying pKS03 and pKS03mut6 was confirmed by RT-PCR using primers SigCRTF and MycRTR, indicating that the mutation introduced had no effect on transcription (Fig. 3c[Fig f3]). pKS03, pKS03mut5 and pKS03mut6 were then transformed into *M. tuberculosis* H37Rv. SigC-Myc was detected from the cell-free extracts of *M. tuberculosis* bearing pKS03 and pKS03mut5 but not pKS03mut6 or pEJMyc (Fig. 3d[Fig f3]). The presence of *sigC-myc* transcript in *M. tuberculosis* expressing pKS03mut6 was confirmed as above (Fig. 3e[Fig f3]). This indicates that translation initiates at the same site in *M. tuberculosis* as in *M. smegmatis*. Therefore, the translational start site for SigC is found between residues 5 and 6, suggesting that the start site annotated for H37Rv is the correct one for this gene.

This finding contradicts a recent report that the longer, CDC1551 annotation was correct for H37Rv SigC (Thakur *et al.*, 2007). As the experiments to determine the SigC translational start site presented above were all performed on mycobacterial strains grown to mid-exponential phase, and the experiments performed by Thakur *et al.* (2007) were performed using cell extracts from stationary-phase bacteria, expression of Myc-tagged SigC was compared at different growth phases, to investigate the possibility that translation of SigC initiates at different sites under different conditions. As well as comparing the size of the tagged protein produced by pKS03, pJS014 was constructed, containing the Myc tag fused in-frame to the codon immediately upstream of the start site identified above. *M. smegmatis* mc^2^155 or *M. tuberculosis* H37Rv containing pEJMyc, pKS03 or pJS014 were grown to either mid-exponential (OD_600_ ∼0.6), late-exponential/early stationary (OD_600_ ∼1.0) or stationary (OD_600_ ∼2.0) phase and cell-free extract was prepared. No SigC-Myc was detected from *M. smegmatis* or *M. tuberculosis* bearing either pEJMyc or pJS014 under any of these conditions, and the SigC-Myc from *M. smegmatis* or *M. tuberculosis* expressing pKS03 was consistently 21.6 kDa (Fig. 3f[Fig f3]). The presence of *sigC-myc* transcript in strains expressing pJS014 was verified by RT-PCR using the primers JORTF and MycRTR. Therefore, we have been unable to detect translation of SigC from a site other than the H37Rv annotated start site.

### Determination of the transcriptional and translational start sites of Rv1955

**Fig. 4. f4:**
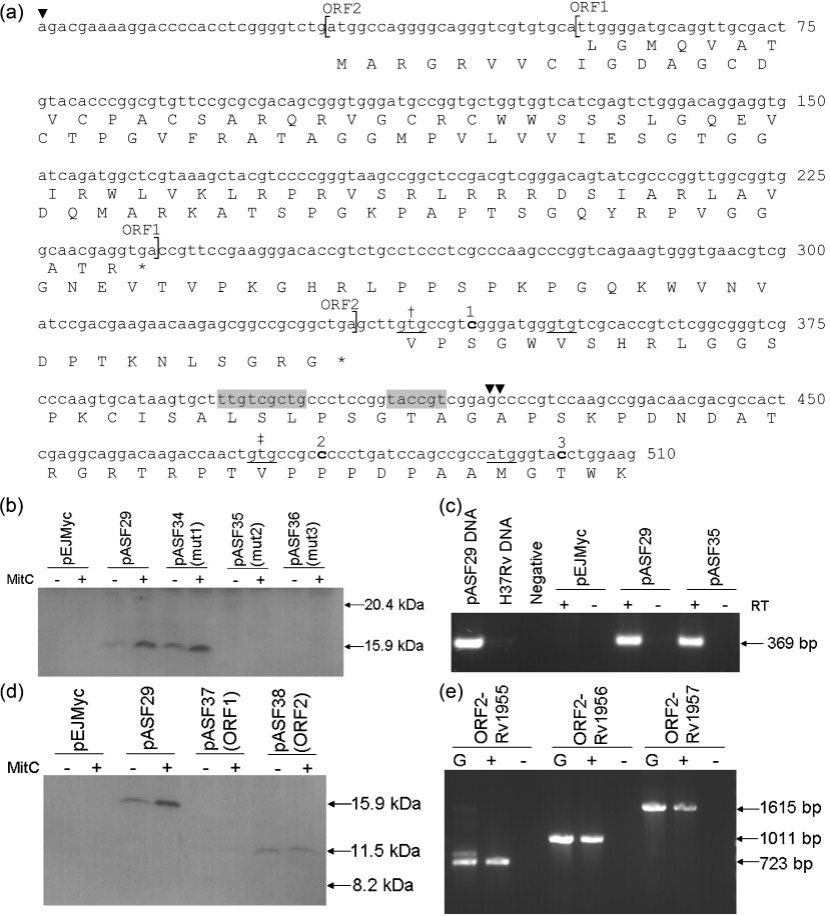
Identification of the transcriptional and translational start sites of Rv1955 and the discovery of a novel ORF upstream of Rv1955. (a) The nucleotide sequence of the beginning of the Rv1955 gene including 336 bp upstream of the annotated start codon. The RecA-NDp motif is shaded and the transcriptional start sites identified by 5′ RACE are shown (▾). Possible start codons are underlined; the annotated start site is indicated by † and the experimentally defined start codon is indicated by ‡. To determine the translational start codon, single-residue deletions (bold and numbered) were created between potential start codons. The location of two potential ORFs upstream of Rv1955 are indicated with square brackets, and their amino acid translations are given. (b) Western blot analysis of cell-free extracts of *M. tuberculosis* containing pEJMyc, pASF29, pASF34 (containing mutation 1), pASF35 (mutation 2) or pASF36 (mutation 3), from cultures with and without the addition of mitomycin C (MitC). A 15.9 kDa band corresponding to Rv1955-Myc can be detected from strains containing pASF29 and pASF34 but not pEJMyc, pASF35 or pASF36. The effect of mitomycin C-induced DNA damage on expression levels of Rv1955-Myc can be seen. (c) RT-PCR analysis of RNA extracts of *M. tuberculosis* containing pEJMyc, pASF29 or pASF35. A 369 bp band indicating the presence of Rv1955-*myc* transcript was detected from strains containing pASF29 and pASF35 but not from strains containing pEJMyc or the RNA without RT controls. (d) Western blot analysis of cell-free extracts of *M. tuberculosis* containing pEJMyc, pASF29, pASF37 (ORF1) or pASF38 (ORF2), from cultures with and without the addition of mitomycin C. Again a 15.9 kDa band corresponding to Rv1955-Myc can be detected from the strain containing pASF29. A potential 8.2 kDa ORF1-Myc protein was undetectable from pASF37. A 11.5 kDa band corresponding to ORF2-Myc can be detected from the strain containing pASF38 but not from strains containing pASF28, pASF37 or pEJmyc. (e) RT-PCR analysis of an *M. tuberculosis* H37Rv RNA extract shows that ORF2 is co-transcribed with Rv1955–Rv1957. The 723 bp, 1011 bp and 1615 bp bands indicate the presence of ORF2-Rv1955, ORF2-Rv1956 and ORF2-Rv1957 transcripts within cDNA from *M. tuberculosis* H37Rv (G, genomic DNA positive control; +, RT positive cDNA; −, RT negative control).

The function of the *M. tuberculosis* H37Rv protein Rv1955 is unknown but the gene is thought to be co-transcribed with its two downstream genes, Rv1956, encoding a possible transcriptional regulatory protein, and Rv1957, encoding another protein of unknown function (Cole *et al.*, 1998; Rand, 2003). A promoter motif, thought to regulate a LexA/RecA-independent DNA damage response mechanism in *M. tuberculosis* (designated RecA-NDp, for RecA non-dependent promoter), has been identified approximately 70 bp downstream of the Rv1955 annotated translational start site, suggesting that the predicted annotation is incorrect (Gamulin *et al.*, 2004). Transcriptional start site mapping using 5′ RACE identified two sites for Rv1955, at either 85 or 86 bp downstream and at 336 bp upstream of the annotated translation start site, suggesting that two promoters (designated P1 and P2 respectively) control the expression of the Rv1955–Rv1957 operon (Fig. 4a[Fig f4]). As RACE uses a poly-cytosine tail and the complementing strand was sequenced, sites cannot be precisely mapped where transcription may begin at a guanine residue; hence the approximate location of the P1 promoter transcriptional start site. The position of the RecA-NDp motif corresponds with this shorter transcript, further supporting implications that this protein may be smaller than expected. We therefore decided to confirm the *M. tuberculosis* Rv1955 translational start site experimentally.

The Rv1955 gene and its upstream region, including both promoters, was cloned into pEJMyc to give pASF29, containing Rv1955 with an in-frame C-terminal Myc tag. Rv1955 contains three alternative start codons downstream of the P1 promoter and annotated start site. Single base pair deletions were created downstream of the annotated start site and each potential start codon subsequent to P1 within pASF29 to give plasmids pASF34, pASF35 and pASF36, where residues 1–3 were deleted respectively (Fig. 4a[Fig f4]). Rv1955-Myc was undetectable from all extracts of *M. smegmatis* mc^2^155 containing pASF29 or its derivatives (data not shown). The plasmids were therefore transformed into *M. tuberculosis* H37Rv and, in an attempt to increase Rv1955-Myc expression levels, cell-free extract was prepared from cultures with mitomycin C-induced DNA damage alongside uninduced control cultures. Upon Western blotting, Myc-tagged Rv1955 was expected to be detected between 20.4 kDa and 15.1 kDa, for the largest and smallest potential ORFs, respectively. Rv1955-Myc was detected in extracts from uninduced cultures, and to a greater extent in induced cultures, from *M. tuberculosis* containing pASF29 and pASF34 at 15.9 kDa, but was not detected in either extract from *M. tuberculosis* containing pEJMyc, pASF35 or pASF36 (Fig. 4b[Fig f4]). The presence of mRNA transcript for Rv1955-*myc* was confirmed by RT-PCR using primers Rv1955RTF and MycRTR, indicating that the mutations introduced had no effect on transcription (Fig. 4c[Fig f4]). This shows that the translational start site for Rv1955 is between residues 1 and 2, indicating that Rv1955 is not translated from the annotated start site. Furthermore, the translational start cannot be upstream of the transcription start site, so translation must begin from the GTG start codon 135 bp downstream of that annotated. The increased expression level of Rv1955-Myc upon the addition of mitomycin C supports the observation that the predicted DNA damage inducible promoter RecA-NDp controls Rv1955-Rv1957 expression.

### Identification of a novel ORF upstream of Rv1955

As the Rv1955 translational start site was determined to be 135 bp downstream of the annotated start site, there is a 471 bp region between the furthest upstream transcriptional start site and the Rv1955 translational start site. This 471 bp region was therefore analysed for the presence of any potential ORFs. Two possible ORFs were identified and pASF29 was mutated to bring these potential ORFs into frame with the Myc tag, to give pASF37 and pASF38 for ORF1 and ORF2 respectively (Fig. 4a[Fig f4]). pASF37 and pASF38 were transformed into *M. tuberculosis* H37Rv and cell-free extract was prepared from cultures with and without mitomycin C-induced DNA damage. Upon Western blotting, ORF1-Myc and ORF2-Myc were expected to be detected at a maximum of 8.2 kDa and 11.5 kDa respectively. ORF1-Myc was undetectable in both extracts of *M. tuberculosis* containing pASF37 (Fig. 4d[Fig f4]). However, ORF2-Myc was detected in extracts from the uninduced culture, and to a similar extent in the induced culture, from *M. tuberculosis* containing pASF38, but was not detected in either extract from *M. tuberculosis* containing pEJMyc (Fig. 4d[Fig f4]). We have therefore demonstrated that a small, non-annotated ORF upstream of Rv1955 is expressed as a protein within *M. tuberculosis* H37Rv.

Searching the protein database revealed no conserved domains to indicate a possible function for ORF2, nor any significant sequence homology to other bacterial proteins using blastp (Altschul *et al.*, 1990). The expression level of ORF2-Myc does not appear to increase upon the addition of mitomycin C, suggesting that the P2 promoter is not DNA damage inducible. To determine whether ORF2 is co-transcribed with Rv1955–Rv1957, RT-PCR was performed on cDNA generated from *M. tuberculosis* H37Rv RNA using primers spanning the region from ORF2 to Rv1955, Rv1956 and Rv1957 individually. Specific transcripts were detected using all three primer pairs, indicating that ORF2 is part of the Rv1955–Rv1957 operon (Fig. 4e[Fig f4]).

## DISCUSSION

The annotation of bacterial genomes is performed using mathematical models, often with comparison to other genomes and protein databases. As the available information increases these predictions become more accurate; however, not all ORF predictions can be assumed to be correct. This is particularly true of the translational start site, which tends to be more difficult to predict. The initial annotation of ORFs in the *M. tuberculosis* H37Rv genome identified 3974 potential genes (Cole *et al.*, 1998). Reannotation 4 years later led to the identification of 82 new potential genes and the lengths of 60 ORFs being altered, the majority of which were altered at the N-terminus (Camus *et al.*, 2002). We suspected that the translational start sites for a number of *M. tuberculosis* H37Rv genes remain incorrectly annotated, and so we devised a simple approach for determining translational start sites using a combination of epitope tagging and frameshift mutagenesis. Using this assay we were able to determine the translational start sites of three proteins. Of these, LexA was shown to start upstream of the annotated start site, the H37Rv annotated start site was confirmed for SigC, and Rv1955 was shown to start downstream of the annotated start site. We were also able to identify that a previously unrecognized ORF upstream of Rv1955 is expressed as a protein.

The negative regulator LexA is one of the key players in the SOS response, a bacterial response to DNA damage, and homologues have been characterized from both *M. tuberculosis* and *M. smegmatis* (Davis *et al.*, 2002; Durbach *et al.*, 1997; Movahedzadeh *et al.*, 1997). LexA represses transcription of DNA damage inducible genes by binding to an upstream DNA sequence termed the SOS box. DNA damage activates RecA, which in turn stimulates autocatalytic cleavage of LexA, lifting repression of the regulated genes. The initial annotation of the *M. tuberculosis* LexA identified the translational start site based on comparison to the identified LexA sequence from other bacterial species (Movahedzadeh *et al.*, 1997). Since then a number of mycobacterial genomes have been sequenced, the annotation of which in many cases shows a longer N-terminal sequence for LexA, leading us to re-evaluate the *M. tuberculosis* annotation. We were able to demonstrate that the translational start site of LexA is 57 bases upstream of the annotated start, at the same location as the transcriptional start site. Such leaderless mRNAs, with no 5′ untranslated regions, are an increasingly recognized phenomenon and are particularly prevalent in Gram-positive bacteria and archaea (Moll *et al.*, 2002). This alternative translational start site is also likely to apply in *M. bovis* as, although it is annotated to start at a similar site to LexA of *E. coli* and *B. subtilis* (Fig. 1), the N-terminal nucleotide sequence is identical to that of *M. tuberculosis* (data not shown).

Bacterial sigma factors are components of RNA polymerase that bind to specific DNA promoter regions, thus directing the transcription of specific genes. Sigma factor C (SigC) is an extracytoplasmic function sigma factor; this group of sigma factors is involved in the response to stress conditions (Rodrigue *et al.*, 2006). Although the stimulus for SigC activation is not known it has been predicted to be involved in virulence as it is present in the genomes of *M. tuberculosis* and *M. leprae* but absent from the non-pathogenic strain *M. smegmatis* (Cole *et al.*, 1998, 2001; Waagmeester *et al.*, 2005). Indeed, SigC has been shown to play a role in pathogenesis in mouse and guinea pig models of *M. tuberculosis* infections (Karls *et al.*, 2006; Sun *et al.*, 2004). The annotation for *sigC* in two *M. tuberculosis* strains differed significantly in the 5′ region, with *sigC* from strain CDC1551 starting 378 nucleotides further upstream than the gene from strain H37Rv (Camus *et al.*, 2002; Fleischmann *et al.*, 2002). We used our assay to confirm that the annotated start site for H37Rv was correct for this strain. It is likely that the H37Rv annotation is also correct for strain CDC1551, as the sequences over this region are identical. Furthermore, the translational apparatus is highly conserved in these two strains, with the sequences of the majority of ribosomal and initiation factor genes being identical, making translation from an alternative site unlikely. This contradicts a recent investigation, in which the size of SigC present in cell-free extracts from H37Rv was compared to recombinant protein by Western blotting, leading to the conclusion that the CDC1551 annotation was correct (Thakur *et al.*, 2007). We therefore investigated the possibility that translation initiates from different sites under different growth conditions. Such overlapping genes are well documented in eukaryotes and viruses, and have also been identified in several species of bacteria including *E. coli*, *Bacillus subtilis* and *Corynebacterium flavum* (Chen & Paulus, 1988; Follettie *et al.*, 1993; Normark *et al.*, 1983; Plumbridge *et al.*, 1985). However, we were unable to find any evidence for alternative translation initiation in the *sigC* gene in this investigation.

The predicted transcriptional regulator Rv1956 forms an operon with two genes of unknown function, Rv1955 and Rv1957 (Cole *et al.*, 1998; Rand, 2003). Gene expression analyses indicate that the Rv1955–Rv1957 operon is upregulated when *M. tuberculosis* is exposed to heat shock (Stewart *et al.*, 2002), nutrient starvation (Betts *et al.*, 2002) and DNA damage (Rand *et al.*, 2003). The association of the RecA-NDp promoter motif with Rv1955 suggests that this operon may form part of the LexA/RecA-independent response to DNA damage (Gamulin *et al.*, 2004). As the RecA-NDp promoter was found to be within the annotated translated region of Rv1955 we determined the transcriptional and translational start sites of Rv1955 experimentally. Two transcriptional start sites were identified for Rv1955, one downstream and the other upstream of the annotated translation start site, suggesting that two promoters (designated P1 and P2 respectively) control the expression of the Rv1955–Rv1957 operon. The translational start site was found to be 135 bp downstream of the annotated start site and is downstream of both the RecA-NDp motif and the two identified transcriptional start sites. This, along with the observation that Rv1955 is more highly expressed after mitomycin C induction, supports the notion that this operon may form part of the LexA/RecA-independent DNA damage response.

In this investigation we were also able to use the C-terminal Myc tag to identify that a novel ORF located upstream of Rv1955 is expressed as a protein. We suggest that this new ORF be renamed Rv1954A, in keeping with current naming consensus (Camus *et al.*, 2002). Rv1954A does not contain any conserved protein domains and its function remains unknown. Small proteins often remain unannotated within genome annotations as the methods used often exclude potential ORFs below a certain length. The initial *M. tuberculosis* H37Rv annotation limited ORF length to 100 aa (Cole *et al.*, 1998) and reannotation reduced this to 60 aa, resulting in the identification of many more potential ORFs (Camus *et al.*, 2002). Rv1954A is 100 aa in length but was not identified in either annotation. This is probably because Rv1954A overlaps with Rv1954c, which is located on the opposite strand (Cole *et al.*, 1998). However there is no experimental evidence that Rv1954c is expressed as a protein, and this gene is not annotated in the *M. tuberculosis* strain CDC1551 genome (Fleischmann *et al.*, 2002), so it may represent a mis-annotation in H37Rv.

The Rv1954A gene lies between the two identified transcriptional start sites of Rv1955. Rv1954A is therefore only expressed from the P2 promoter, which, unlike the P1 promoter, does not appear to be DNA damage inducible. After RT-PCR analysis it appears that Rv1954A–Rv1957 form a single operon and that the P2 promoter controls the expression of all these proteins under the growth conditions used in the study, and perhaps upon the addition of an unknown stimulus. The P1 promoter also appears to control the expression of Rv1955–Rv1957 upon DNA damage.

In conclusion, we have devised a simple method for determining the translational start sites of predicted ORFs and demonstrated its use on a number of mycobacterial proteins. We have also used this method to identify a previously unannotated ORF, Rv1954A. For many ORFs, but not all, it is possible to perform this assay in *M. smegmatis*, a model organism for *M. tuberculosis,* which grows faster than *M. tuberculosis* and is easier to work with as it is an ACDP containment level 1 organism. This may be the case even if the gene is not present in the *M. smegmatis* genome, as homologues of many of the regulatory factors required for the translation of *M. tuberculosis* genes are conserved between *M. tuberculosis* and *M. smegmatis* (Tyagi & Sharma, 2002). For example, we were able to detect SigC-Myc even though no native SigC is present in the *M. smegmatis* genome (Waagmeester *et al.*, 2005). It is likely that the translation initiation sites and new ORFs outlined in this paper also apply to other members of the *M. tuberculosis* complex.

## Figures and Tables

**Table 1. t1:** Plasmids used in this study

**Name**	Description and construction*	**Reference**
pMV306	Integrating mycobacterial cloning vector	Stover *et al.* (1991)
pEJ410	Deletion of part of polylinker between *Xba*I and *Mlu*I in pMV306	This study
pEJMyc	Myc tag vector; insertion of annealed oligonucleotides mycF and mycR into pEJ410 between *Eco*RI and *Eco*RV	This study
pKS03	pEJMyc containing SigC and 644 bp upstream of H37Rv annotated start; insertion of 1199 bp PCR product from primers SigCupclone and SigCdownclone into *Hin*dIII and *Eco*RV	This study
pKS03mut2	pKS03 derivative missing residue 2†; SDM using primers SigCmut2F and SigCmut2R	This study
pKS03mut3	pKS03 derivative missing residue 3†; SDM using primers SigCmut3F and SigCmut3R	This study
pKS03mut4	pKS03 derivative missing residue 4†; SDM using primers SigCmut4F and SigCmut4R	This study
pKS03mut5	pKS03 derivative missing residue 5†; SDM using primers SigCmut5F and SigCmut5R	This study
pKS03mut6	pKS03 derivative missing residue 6†; SDM using primers SigCmut6F and SigCmut6R	This study
pJS014	pEJMyc containing 654 bp region upstream of H37Rv SigC annotated start site; insertion of 654 bp PCR product from primers SigCupclone and RSigC into *Hin*dIII and *Xba*I	This study
pKS04	pEJMyc containing LexA and 318 bp upstream of annotated start; insertion of 968 bp PCR product from primers LexAF and LexAR into *Hin*dIII and *Eco*RI	This study
pKS04mut1	pKS04 derivative missing residue 1†; SDM using primers LexAmut1F and LexAmut1R	This study
pASF29	pEJmyc containing Rv1955 and 490 bp upstream of annotated start; insertion of 1006 bp PCR product from primers Rv1955mycF and Rv1955mycR into *Pvu*II and *Xba*I	This study
pASF34	pASF29 derivative missing residue 1†; SDM using primers mycFS1F and mycFS1R	This study
pASF35	pASF29 derivative missing residue 2†; SDM using primers mycFS2F and mycFS2R	This study
pASF36	pASF29 derivative missing residue 3†; SDM using primers mycFS3F and mycFS3R	This study
pASF37	pASF29 derivative where ORF1 is in-frame with C-terminal Myc tag; SDM using primers mycORF1F and mycORF1R	This study
pASF38	pASF29 derivative where ORF2 is in-frame with C-terminal Myc tag; SDM using primers mycORF2F and mycORF2R	This study

*SDM, site-directed mutagenesis. †For location of missing residues see Fig. 2(a), 3(a) or 4(a).

**Table 2. t2:** Primers used in this study

**Name**	**Sequence 5′–3′**
MycF	AATTCGCGGCCGATATCTAGAACAAAAACTCATCTCAGAAGAG
MycR	CAGATCCTCTTCTGAGATGAGTTTTTGTTCTAGATATCGGCCGCG
LexAF	AGAAAGCTTCCGGTGTCATGTTCGCTCCT
LexAR	AGAGAATTCATGACCTTGCGGATCACCGTGAC
LexAmut1F	CGACTACATTCATTGCCATGAAGACAGCAACGACACCTCGGTTGC
LexAmut1R	GCAACCGAGGTGTCGTTGCTGTCGTTCATGGCAATGAATGTAGTCG
SigCupclone	GCTGCGGAGGAAGCTTGTAAATGCCGCGGTG
SigCdownclone	ATCCGCCGGTGAGGTCGTCGGGCTCCGCGTC
RSigC	TCTCTAGAGCGGGCATAAACCATAGCCCGGCAGG
SigCmut2F	CGCTCCGGCCGGCAAGGTTGCGATGCCGGACGGGCCCCGC
SigCmut2R	GCGGGGCCCGTCCGGCATCGCAACCTTGCCGGCCGGAGCG
SigCmut3F	GCCGTAGTATCACACGCGCGAGATGGCAGGCGCCAAAGCG
SigCmut3R	CGCTTTGGCGCCTGCCATCTCGCGCGTGTGATACTACGGC
SigCmut4F	GTATCACACGCGCGCAGATGCAGGCGCCAAAGCGCATTCG
SigCmut4R	CGAATGCGCTTTGGCGCCTGCATCTGCGCGCGTGTGATAC
SigCmut5F	GGCTATGGTTTATGCCCATGCCGCGACGGCAAGCGACGAC
SigCmut5R	GTCGTCGCTTGCCGTCGCGGCATGGGCATAAACCATAGCC
SigCmut6F	GGCTATGGTTTATGCCATGACCGCGACGGC
SigCmut6R	GCCGTCGCGGTCATGGCATAAACCATAGCC
Rv1955 GSP1	ATCGCAACTCCAGAATGTC
Rv1955 GSP2	AATACCGGCCGGCCATCCACAG
Rv1955 GSP3	TGGATCAGGGGGCGGCACAG
Rv1955mycF	GTACAGCTGCGCACCGACCCGACCCAGA
Rv1955mycR	AGCTCTAGACCGATCGGTGGGGTGTCGCC
mycFS1F	CGCGGCTGAGCTTGTGCCGTGGGATGGGTGTCGCACCGTC
mycFS1R	GACGGTGCGACACCCATCCCACGGCACAAGCTCAGCCGCG
mycFS2F	GACAAGACCAACTGTGCCGCCCCTGATCCAGCCGCCATGG
mycFS2R	CCATGGCGGCTGGATCAGGGGCGGCACAGTTGGTCTTGTC
mycFS3F	GATCCAGCCGCCATGGGTACTGGAAGTTCTTCCGGGCATC
mycFS3R	GATGCCCGGAAGAACTTCCAGTACCCATGGCGGCTGGATC
mycORF1F	GGTTGGCGGTGGCAACGAGGGAACAAAAACTCATCTCAG
mycORF1R	CTGAGATGAGTTTTTGTTCCCTCGTTGCCACCGCCAACC
mycORF2F	GAACAAGAGCGGCCGCGGCGAACAAAAACTCATCTCAG
mycORF2R	CTGAGATGAGTTTTTGTTCGCCGCGGCCGCTCTTGTTC
MycRTR	CCTCTTCTGAGATGAGTTTTTG
SigCRTF	TCGAGGTAACCACGATGATC
LexARTF	CCACCGTCAAGACGTTCAAA
Rv1955RTF	AGTTCTTCCGGGCATCTGT
JORTF	TTGCGCATGCCGTAGTATCA
ORF2F	TGCCGGTGCTGGTGGTCATC
ORF2-Rv1955R	GTCGCCGAAGGCTCTTTTCCAG
ORF2-Rv1956R	TGGGGTCGCTGGGTTCCTTCT
ORF2-Rv1957R	GGCGGCGTATGCCGTAAGTTCT

## Abbreviations

- ACDP, Advisory Committee on Dangerous Pathogens
- RACE, rapid amplification of cDNA ends
- SDM, site-directed mutagenesis
